# Is Patent “Evergreening” Restricting Access to Medicine/Device Combination Products?

**DOI:** 10.1371/journal.pone.0148939

**Published:** 2016-02-24

**Authors:** Reed F. Beall, Jason W. Nickerson, Warren A. Kaplan, Amir Attaran

**Affiliations:** 1 Population Health Program, Faculty of Medicine and Faculty of Law, University of Ottawa, Ottawa, Ontario, Canada; 2 Bruyère Research Institute, Ottawa, Ontario, Canada; 3 Center for Global Health & Development, Department of Global Health, WHO Collaborating Center for Pharmaceutical Policy, Boston University School of Public Health, Boston, Massachusetts, United States of America; Research Center Borstel, GERMANY

## Abstract

**Background:**

Not all new drug products are truly new. Some are the result of marginal innovation and incremental patenting of existing products, but in such a way that confers no major therapeutic improvement. This phenomenon, pejoratively known as “evergreening”, can allow manufacturers to preserve market exclusivity, but without significantly bettering the standard of care. Other studies speculate that evergreening is especially problematic for medicine/device combination products, because patents on the device component may outlast expired patents on the medicine component, and thereby keep competing, possibly less-expensive generic products off the market.

**Materials and Methods:**

We focused on four common conditions that are often treated by medicine/device product combinations: asthma and chronic obstructive pulmonary disease (COPD), diabetes, and severe allergic reactions. The patent data for a sample of such products (n = 49) for treating these conditions was extracted from the United States Food and Drug Administration’s Orange Book. Additional patent-related data (abstracts, claims, etc) were retrieved using LexisNexis TotalPatent. Comparisons were then made between each product’s device patents and medicine patents.

**Results:**

Unexpired device patents exist for 90 percent of the 49 medicine/device product combinations studied, and were the only sort of unexpired patent for 14 products. Overall, 55 percent of the 235 patents found by our study were device patents. Comparing the last-to-expire device patent to that of the last-to-expire active ingredient patent, the median additional years of patent protection afforded by device patents was 4.7 years (range: 1.3–15.2 years).

**Conclusion:**

Incremental, patentable innovation in devices to extend the overall patent protection of medicine/device product combinations is very common. Whether this constitutes “evergreening” depends on whether these incremental innovations and the years of extra patent protection they confer are proportionately matched by therapeutic improvements in the standard of care, which is highly debatable.

## Introduction

Many medicines are inextricably paired with specific devices for administering them. Asthma, chronic obstructive pulmonary disease (COPD), diabetes, and severe allergic reactions are examples of common conditions that, depending on the medicine prescribed, may come with a proprietary inhaler or injector that is integrated into the product design.

What may not be obvious is that in these combinations, the medicine and the device are separately patentable. Thus, even after all the patents on the medicine expire, remaining patents on the associated device, or parts thereof, can prevent generic competitors from emerging. In other words, medicine patents and device patents are not coextensive, but synergistic—a relationship companies can use strategically to prolong market exclusivity [1].

For example, in 2008 when the United States’ Food and Drug Administration (USFDA) mandated that all metered-dose inhalers stop using ozone-depleting chlorofluorocarbon (CFC) propellants, pharmaceutical companies switched to hydrofluoroalkane (HFA) propellants [2]. Albuterol, for example, is a common medication prescribed for both asthma and COPD, and is old and off-patent, but new HFA-compatible valves, elastomers, and surfactants were needed to comply with new FDA requirements, resulting in new patentable devices [3]. The new, proprietary HFA-based albuterol-containing products entered the market at double or triple the price of the old, generic CFC-based products [4]. Similar price movements have been observed for human insulin, which despite being an old and off-patent has no generic competition in the United States [5].

The patent system’s *raison d'être* is based, however imperfectly, on the social bargain that the market exclusivity of a 20-year patent incents and rewards worthwhile inventions. It is therefore debatable whether incremental device innovation and patenting creates proportionate therapeutic improvements (or environmental improvements, in the case of HFA-based inhalers) [6–9]. The term “evergreening” has been coined to disparage the practice of making incremental, patentable innovations for medicines without corresponding benefit, particularly if patients are aggressively or forcibly transitioned to the new product: examples include omeprazole versus esomeprazole, or memantine versus memantine extended release [10]. This paper explores the possible salience of evergreening to devices, which has received less attention [1, 5, 9–11].

This is the first study known to the authors to analyze the effect of medicine/device patent synergies in several therapeutic areas where they prevail: inhaled medicines for treating asthma and COPD, auto-injectors for treating anaphylaxis, and insulin pens for the treatment of type 1 (insulin dependent) diabetes. We test the hypothesis that having two modes of patenting, rather than one, prolongs product exclusivities. Where this is the case, it can negatively affect health by precluding competition that reduces prices, as is anecdotally observed. Our study explores the mechanism whereby this can happen.

## Materials and Methods

Products such as auto-inject pens or inhalers sold pre-loaded with the active ingredient qualify for inclusion on the USFDA’s “Orange Book” because that regulator considers them inseparable from the medicines that contain them. The Orange Book is an online database that lists the United States patent holdings of most medicines with FDA marketing approval. The Orange Book however has limitations: it only includes devices primarily associated with the medicine, and not necessarily those secondarily associated (e.g., insulin pumps or continuous glucose monitors) [1].

We identified and included all insulin or epinephrine device products in the Orange Book [12] by searching for the name of the active ingredient, regardless of patent status. For our sample of asthma and COPD medicines, we located all patent data in the Orange Book for all products on the FDA’s list of asthma and COPD medicines [13]. After compiling the Orange Book patent data (including patent numbers and expiration dates), additional information on each of these patents (titles, abstracts, claims, etc) was extracted from the LexisNexis’ Total Patent® database [14]. This produced lists of patents pertaining to the medicine and device components of each combination product.

We categorized each patent by its applicability to the device or to the compound. The expiration date of the last-to-expire (co-) formulation or compound patent on the active ingredient was then identified and compared to the expiration date of its last-to-expire device patent. Using the Orange Book, we further documented the number of suppliers offering an equivalent medicine/device combination (i.e., one with an identical active ingredient, formulation, strength, and route of administration) in order to provide an impression of the level of exclusivity within these markets.

## Results

Our main results are detailed in Table 1 (the actual data are available in S1 File). All of the results outlined in this section are based on these data. Our search returned a total of 49 medicine/device combination products—32 inhalers containing an asthma or COPD medicine, 3 pens containing epinephrine, and 14 products involving an insulin-containing device, either a pen or inhaler.

**Table 1:** Drug/device combination products compared by patent type (device or medicament) and last patent expiration.

Product	Active Ingredient	Device patent count	Non-device patents count	Proportion of device versus medicine patents	Last medicine patent expiration*	Last device patent expiration*	Patent protection added by device patent (in years)**	Number of suppliers of a marketed equivalent (brand or generic)***	Outcome typology****
**COPD inhalers**									
Tudorza Pressair	ACLIDINIUM BROMIDE	3	4	43%	07-Aug-22	22-Apr-27	4.7	1	Device extension
ProAir HFA	ALBUTEROL SULFATE	2	3	40%	12-Sep-23	07-Sep-28	5.0	3	Device extension
ProAir Respiclick	ALBUTEROL SULFATE	8	0	100%	n/a	26-Mar-28	12.6	1	Only device patents
Proventil HFA	ALBUTEROL SULFATE	2	0	100%	n/a	28-Dec-16	1.4	3	Only device patents
Ventolin HFA	ALBUTEROL SULFATE	15	2	88%	01-Dec-21	19-Dec-23	2.0	3	Device extension
Combivent Respimat	ALBUTEROL SULFATE; IPRATROPIUM BROMIDE	17	0	100%	n/a	13-Mar-28	12.6	1	Only device patents
Duoneb	ALBUTEROL SULFATE; IPRATROPIUM BROMIDE	1	0	100%	n/a	28-Dec-21	6.4	6	Only device patents
Brovana	ARFORMOTEROL TARTRATE	0	12	0%	09-Nov-21	n/a	n/a	1	No device patents
QVAR	BECLOMETHASONE DIPROPIONATE	2	1	67%	07-Jul-15	13-Mar-18	2.7	1	Device extension
Pulmicort Flexhaler	BUDESONIDE	4	0	100%	n/a	08-May-18	2.7	1	Only device patents
Symbicort Turbuhaler	BUDESONIDE; FORMOTEROL FUMARATE DIHYDRATE	8	4	67%	29-Jan-23	07-Apr-29	6.2	1	Device extension
Alvesco	CICLESONIDE	2	5	29%	13-May-18	28-Dec-16	n/a	1	Old device
Aerospan HFA	FLUNISOLIDE	0	1	0%	07-Jul-15	n/a	n/a	1	No device patents
Breo Ellipta	FLUTICASONE FUROATE; VILANTEROL TRIFENATATE	7	7	50%	03-Aug-21	11-Oct-30	9.2	1	Device extension
Flovent Diskus	FLUTICASONE PROPIONATE	2	0	100%	n/a	23-Aug-16	1.0	1	Only device patents
Flovent HFA	FLUTICASONE PROPIONATE	16	2	89%	01-Dec-21	26-Aug-26	4.7	1	Device extension
Advair Diskus	FLUTICASONE PROPIONATE; SALMETEROL XINAFOATE	1	0	100%	n/a	23-Aug-16	1.0	1	Only device patents
Advair HFA	FLUTICASONE PROPIONATE; SALMETEROL XINAFOATE	16	2	89%	01-Dec-21	26-Aug-26	4.7	1	Device extension
Foradil	FORMOTEROL FUMARATE	2	0	100%	n/a	28-Nov-20	9.2	1	Only device patents
Perforomist	FORMOTEROL FUMARATE	0	5	0%	22-Jun-21	n/a	n/a	1	No device patents
Dulera	FORMOTEROL FUMARATE; MOMETASONE FUROATE	0	5	0%	21-May-20	n/a	n/a	1	No device patents
Arcapta Neohaler	INDACATEROL MALEATE	1	3	25%	10-Oct-20	11-Oct-28	8.0	1	Device extension
Atrovent HFA	IPRATROPIUM BROMIDE	3	2	60%	04-Nov-14	17-Jan-30	15.2	1	Device extension
Xopenex	LEVALBUTEROL TARTRATE	1	2	33%	08-Oct-24	17-Nov-15	n/a	1	Old device
Asmanex	MOMETASONE FUROATE	0	4	0%	27-Aug-17	n/a	n/a	1	No device patents
Asmanex Twisthaler	MOMETASONE FUROATE	4	7	36%	17-Sep-18	20-Aug-17	n/a	1	Old device
Striverdi Respimat	OLODATEROL HYDROCHLORIDE	16	7	70%	12-May-25	13-Mar-28	2.8	1	Device extension
Serevent Diskus	SALMETEROL XINAFOATE	1	0	100%	n/a	23-Aug-16	1.0	1	Only device patents
Spiriva	TIOTROPIUM BROMIDE	1	9	10%	22-Jan-22	12-Mar-27	5.1	1	Device extension
Spiriva Respimat	TIOTROPIUM BROMIDE	16	1	94%	30-Jan-18	13-Mar-28	10.1	1	Device extension
Incruse Ellipta	UMECLIDINIUM BROMIDE	6	4	60%	27-Jul-25	11-Oct-30	5.2	1	Device extension
Anoro Ellipta	UMECLIDINIUM BROMIDE; VILANTEROL TRIFENATATE	7	6	54%	27-Jul-25	11-Oct-30	5.2	1	Device extension
**Epinephrine pens**									
AUVI-Q	EPINEPHRINE	17	0	100%	n/a	02-Nov-29	14.2	2	Only device patents
Epipen	EPINEPHRINE	4	0	100%	n/a	11-Sep-25	10.1	1	Only device patents
Twinject	EPINEPHRINE	2	0	100%	n/a	04-Feb-25	9.5	2	Only device patents
**Insulin pens**									
Novolog Penfill	INSULIN ASPART RECOMBINANT	3	2	60%	20-Dec-17	02-Jun-15	n/a	1	Old device
Novolog Flexpen	INSULIN ASPART RECOMBINANT	3	2	60%	20-Dec-17	21-Jul-21	3.6	1	Device extension
Novolog Flextouch	INSULIN ASPART RECOMBINANT	5	2	71%	20-Dec-17	03-Aug-26	8.6	1	Device extension
Novolog mix Flexpen	INSULIN ASPART PROTAMINE; INSULIN ASPART	3	2	60%	19-Dec-17	21-Jan-21	3.1	1	Device extension
Lantus	INSULIN GLARGINE RECOMBINANT	1	3	25%	23-Jan-24	23-Mar-28	4.2	1	Device extension
Lantus Solostar	INSULIN GLARGINE RECOMBINANT	6	0	100%	23-Jan-24	12-Apr-25	1.2	1	Device extension
Toujeo Solostar	INSULIN GLARGINE RECOMBINANT	7	0	100%	23-Jan-24	23-Mar-28	4.2	1	Device extension
Apidra Solostar	INSULIN GLULISINE RECOMBINANT	7	4	64%	19-Dec-17	23-Sep-27	9.8	1	Device extension
Humalog Kwikpen 100	INSULIN LISPRO RECOMBINANT	1	0	100%	n/a	09-Aug-24	9.0	1	Only device patents
Humalog Kwikpen 200	INSULIN LISPRO RECOMBINANT	1	2	33%	11-Jun-18	09-Aug-24	6.2	1	Device extension
Humalog mix Kwikpen	INSULIN LISPRO PROTAMINE; INSULIN LISPRO	1	0	100%	n/a	09-Aug-24	9.0	1	Only device patents
Afrezza 4	INSULIN RECOMBINANT HUMAN	10	21	32%	08-Mar-31	12-Jul-32	1.3	1	Device extension
Afrezza 8	INSULIN RECOMBINANT HUMAN	9	19	32%	08-Mar-31	12-Jul-32	1.3	1	Device extension
Afrezza 12	INSULIN RECOMBINANT HUMAN	10	14	42%	11-Jun-30	12-Jul-32	2.1	1	Device extension

* Pediatric and other patent extensions are included whenever relevant.

** For medicines listing no patent on the active ingredient, only device patents were listed. We used August 15, 2015 (the date of accessing the database) as the baseline.

***Equivalents were defined as those delivering the identical medicament, strength, (co-)formulation, and route of administration.

****"Device extension" means the last-to-expire device patent was after that of the last-to-expire formulation patent; "Only device patents" means that only device patents remain unexpired and that all patents of any other type have expired; “Old device" means the last-to-expire patent on the formulation was after that of the last-to-expire device patent; and “No device patents" means that no unexpired device patents were listed. Only patents on the formulation remain unexpired.

### Combination product’s device patent portfolios versus other patents

Table 1 lists the number of patents directed to a device versus those directed to the active ingredient alone. Nearly all combination products (44 of 49 products or 90 percent) listed at least one patent on aspects of the delivery device itself. The median, rather than the average (an indicator more sensitive to outliers), combination product had 3 device patents listed and only 2 on the medicament.

Overall, our data extraction from the Orange Book yielded 235 patents. Of these, 55 percent (n = 129) are patents on the device. These outcomes demonstrate that, at least with respect to a simple count of what is listed in the Orange Book, a given combination product in our sample has more patents on the device relative to all other categories.

We further importantly observe that 14 combination products (14/49 or 28.5 percent) only listed device patents (i.e., all patents on the active ingredient have expired or never existed) illustrating that patent activity on the device can continue well beyond the expiration of the patent on the medicine it delivers.

### Length of remaining patent protection afforded by device patents

**Fig 1:**
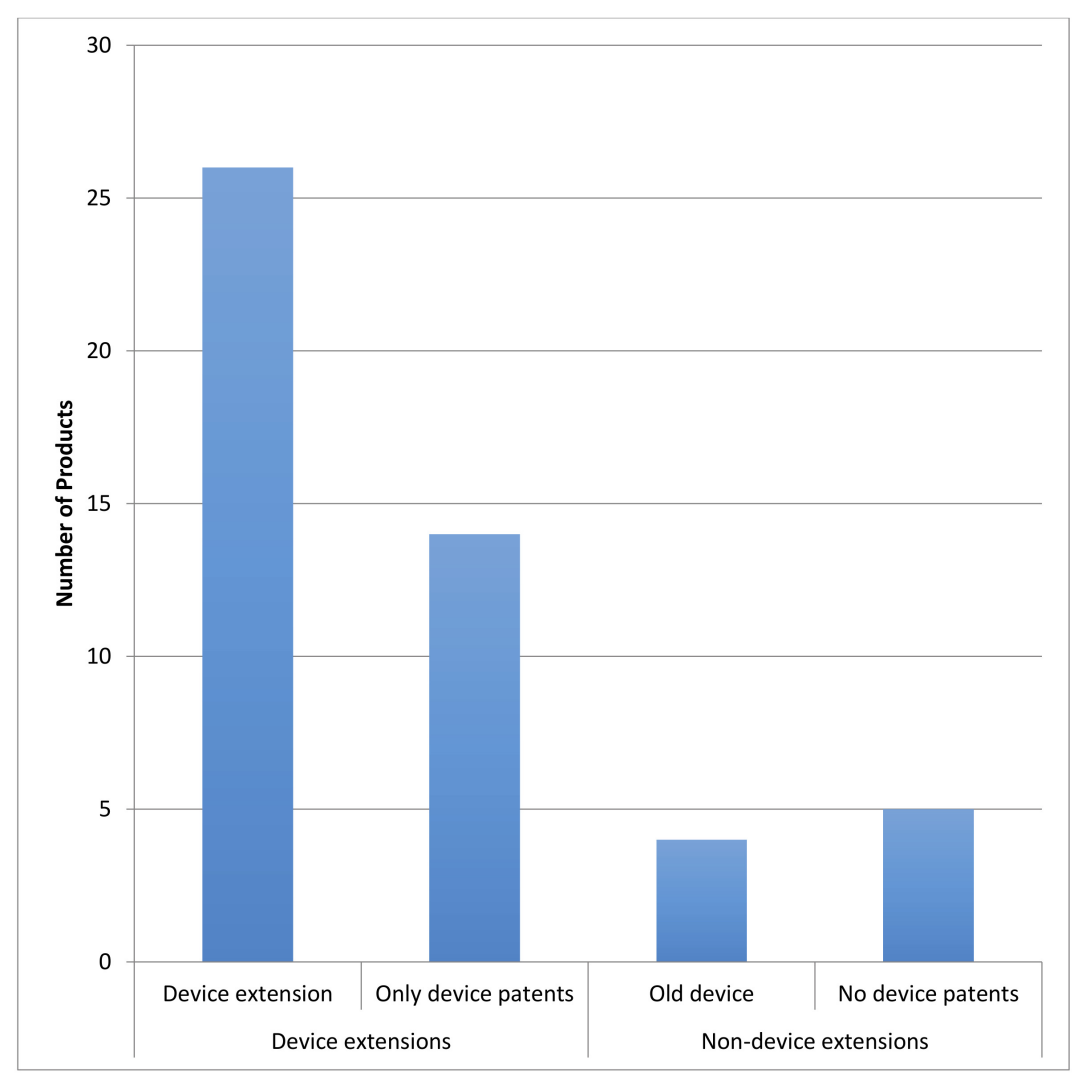
Frequency of patent protection extensions via device patenting amongst the 49 combination products.

Fig 1 depicts the relative frequency of patent protection extensions evident in the 49 products as defined by the outcome typology indicated in the final column of Table 1. Patent protection extensions via the device were the case in 40 of 49 (82 percent) of the combination products investigated.

As for how many years of patent protection was gained through device patenting, two comparisons are possible. The first included those combination products for which both device and medicine patents were listed. This comparison was possible for 35 products in the sample (35/49 or 71 percent). Of these 35 products, 26 had a device patent expiration date that was later than the active ingredient patent expiration date. The median difference between these dates was 4.7 years. The most extreme case was Atrovent HFA®, with 15.2 years between the date of the last-to-expire active ingredient and last-to-expire device patent. The smallest gap was 1.3 years, for the lower dose of Afrezza®, the very new inhaled human insulin product by Sanofi.

The second comparison was for the 14 products for which only device patents were listed. Using the time of writing as the baseline (August 15, 2015), the average combination product had 7.1 years of patent protection remaining by way of the device (an expiration year of 2022). The median number of added years was 9.0 as there were several combination products with large incremental patent life due to the device alone. AUVI-Q®, for example, had a device patent expiring in 2029 (14.2 years) and ProAir Respiclick® had one expiring in 2027 (12.6 years).

### Prevalence of market exclusivity

Because of device patents, none of these combination products included in this study are entirely off-patent. According to the Merck Index (a scientific reference publication that lists patents of therapeutic compounds) [15], 37 percent of the products in our sample have an original compound patent that expired prior to the year 2000 and yet all device/compound combination products in our sample listed at least one unexpired patent.

There was little competition present in the market for these combination products, as each one was typically the only one on the market with its active ingredient(s), (co-) formulation, strength, and route of administration. Table 1 quantifies the number of suppliers carrying an equivalent combination product. Of the 49 combination products analyzed, there was only more than one supplier for formulations of albuterol, albuterol + ipratropium, and epinephrine. Interestingly, in the cases of albuterol and epinephrine, all suppliers were offering their own branded, patented combination product for the same drug formulation.

## Discussion

In our sample, there were more active patents listed, and with later expiration dates, on the devices as opposed to the medicine itself. This result is in part due to the fact that device components are typically developed (and therefore patented) after that of the active ingredient, allowing the patent portfolio of these combination products to extend for several additional years, due to the younger device patent.

Our study demonstrates that medicines for which patents have long expired in the United States can be placed behind a second tier of patent protection for their delivery devices. Whether this is “evergreening”, however, requires consideration of that pejorative term. Underlying any such assessment is the reasoning that the state should only offer patents, which are private rights, in exchange for a proportionate public benefit. However, there is no single, agreed-upon, rigorous definition of evergreening in the literature, so any conclusion in this regard is open to debate. Our results allow for a more nuanced consideration of how the term should be defined in the future, and we propose both general and health-specific definitions here.

In the general definition, evergreening occurs when a secondary patent extends the product’s exclusivity period without a proportionate benefit of any sort. Under this definition, many of the allergy and diabetes products in this study arguably were evergreened by device patents, because there appears to be no therapeutic benefit, but the asthma and COPD inhalers that were originally available with a CFC propellant were not evergreened, because of the environmental benefit in switching from ozone-depleting CFC to safer HFA propellants. (Although with this example, even though there is no therapeutic benefit, there is arguably an ultimate public health benefit of an intact ozone layer).

In the health-specific definition, evergreening occurs when a secondary patent extends the product’s exclusivity period without a proportionate therapeutic benefit. Under this definition, many of the products in this study were arguably evergreened, because the medicines would have the same (or very nearly so) therapeutic benefit if administered by an alternative device. We note, however, that some new devices do offer a therapeutic benefit, for example new MDIs that improve particle deposition, which may allow for a smaller amount of a medicine to be administered [16]. These situations do not neatly fit into our definition of evergreening, but may still allow for prolonged market exclusivity, as subsequent entrants would need to invest in research and development of a new device of comparable efficacy to the existing device with a valid patent, which undoubtedly reduces the profitability of generic versions of the medicine.

Whether the health-specific or the general definition of evergreening is to be preferred is not an appropriate subject for this paper. We do note, however, that one’s view on this distinction is fundamental to agreements or disagreements about the value of device patents such as those studied here. In particular, any government considering legislative action against evergreening (and some already have, such as India) needs to form a clear definition first [17].

When companies can evergreen device-intensive combination products this may foster perverse incentives for originators to, for instance, turn injectable active ingredients into device-intensive combination products or to develop “device-intensive routes” over other options. Given the number of parts on any device, there are multiple independent components to improve and update continuously, opening the door to incremental patenting that presents opportunities for evergreening by layering device patents upon device patents. If left unchecked, the patent system’s weakness for incentivizing patentable ideas rather than the most therapeutically beneficial ones [18] could misdirect research and development dollars into developing new devices when the old ones would do, and/or avoiding more therapeutically beneficial designs that may not be patentable.

Combivent Respimat® is a good example of this. The original patents on the compounds (albuterol and ipratropium) were granted in 1972 and 1970 [15] respectively and have therefore been post-patent for over 25 years. Today these active ingredients are attached to an inhaler that, while appearing a simple device, bears 17 patents with expiration dates ranging from 2014 to 2028. Boehringer, which held the original compound patent on ipratropium granted in 1970, has therefore had patent protection in one form of another on this medicine for 58 years and counting (from 1970–2028).

Without a limit to long patent exclusivities, originators may be able to increase prices without consequences in the absence of competition. They may also be able to progressively phase out old delivery device models, forcing consumers to buy the new and more expensive ones, as has been observed in the media [6]. For example, each one of Novo Nordisk’s line of insulin aspart pens (Novolog Penfill®, Novolog Flexpen®, Novolog Flextouch®) contains a new mechanical feature. In principle, and possibly in fact, the Penfill® model can be phased out and a new one introduced, even if there was no therapeutic lack of efficacy with the Penfill®. In extreme cases, prices may be raised for trivial device improvements with no clinical value. One must wonder whether the health system should absorb the costs that it took to develop a novel “inhaler cap strap” (US patent 8387615) or other similar minor modifications [19].

**Fig 2:**
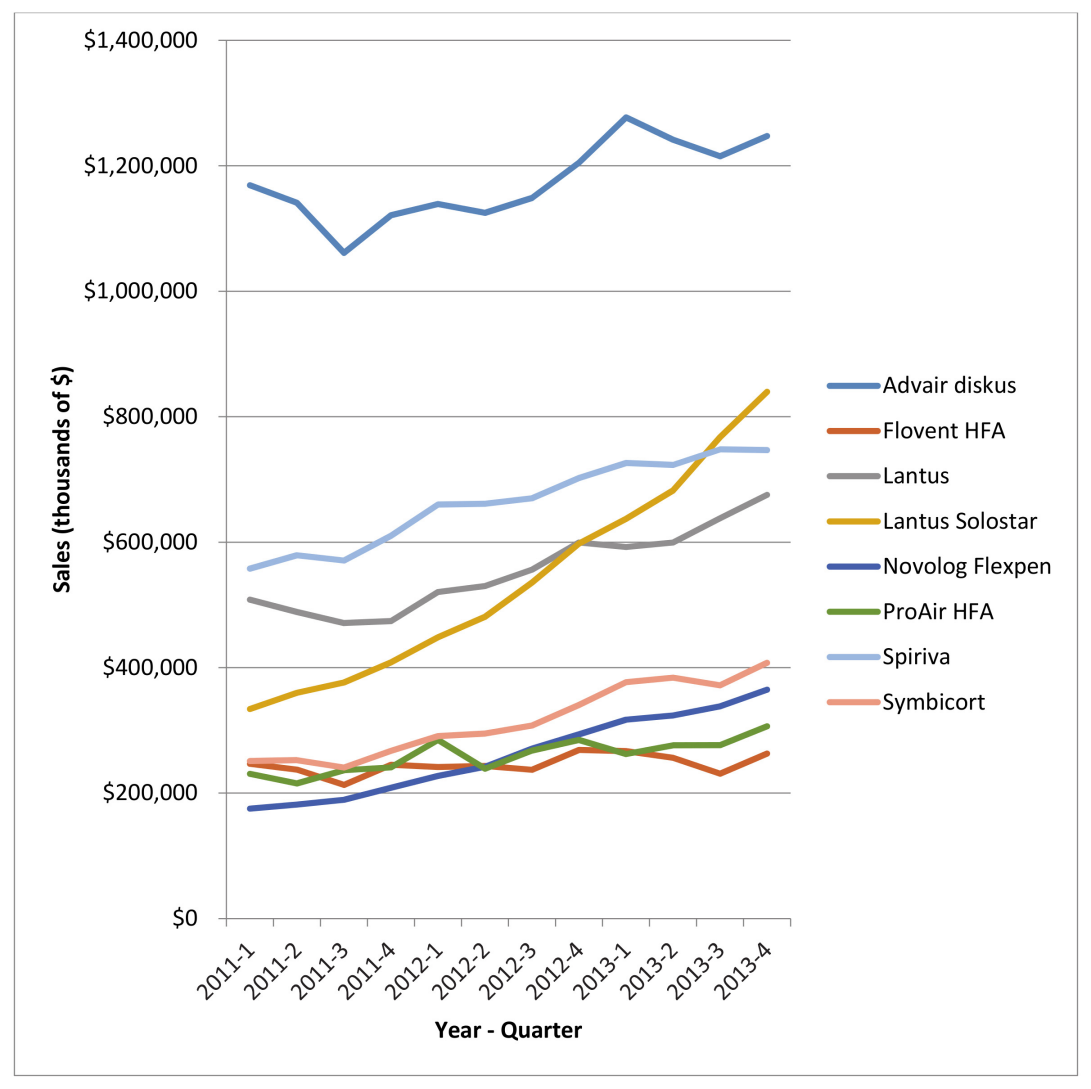
Sales for 8 medication-device combination products in 100-top sellers in the US from 2011–2013 [22].

These considerations are especially urgent considering that many of the 49 combination products are already top sellers in the United States with sales climbing annually. There are three studies referenced by the US National Library of Medicine for pharmaceutical statistics that ranked the top 100 or top 200 drugs by sales [20]. More than one-third (n = 17) of the 49 products examined by this study appeared one or more of those top seller lists [21–23]. Of these, patent protection has been extended through the device in all cases with only a single exception (see Table 2). Fig 2 shows the steady increase of sales from 2011–2013 for the 8 products for which these data were available.

**Table 2:** Top-Selling Drug/device combination products with device extension outcomes.

Product	Active Ingredient	Supplier	Rank in Top 200 by Sales by Pharmacy Times (2012)	Rank in Top-100 Drugs by Sales by Drugs.com (2013)	On Top 200 Drugs by Sales by RxList (2015)	% of device patents in portfolio	Last medicine patent expiration*	Last device patent expiration*	Outcome typology**
**COPD Inhalers**									
ProAir HFA	ALBUTEROL SULFATE	TEVA BRANDED PHARM	53	41	X	40%	12-Sep-23	07-Sep-28	Device extension
Ventolin HFA	ALBUTEROL SULFATE	GLAXOSMITHKLINE	93	95	X	88%	01-Dec-21	19-Dec-23	Device extension
Combivent Respimat	ALBUTEROL SULFATE; IPRATROPIUM BROMIDE	BOEHRINGER INGELHEIM	51	83		100%	15-Aug-15	13-Mar-28	Only device patents
QVAR	BECLOMETHASONE DIPROPIONATE	TEVA BRANDED PHARM	170			67%	07-Jul-15	13-Mar-18	Device extension
Symbicort Turbuhaler	BUDESONIDE; FORMOTEROL FUMARATE DIHYDRATE	ASTRAZENECA	43	31	X	67%	29-Jan-23	07-Apr-29	Device extension
Flovent HFA	FLUTICASONE PROPIONATE	GLAXO GRP LTD	60	58	X	89%	01-Dec-21	26-Aug-26	Device extension
Advair Diskus	FLUTICASONE PROPIONATE; SALMETEROL XINAFOATE	GLAXO GRP LTD	4	5	X	100%	15-Aug-15	23-Aug-16	Only device patents
Advair HFA	FLUTICASONE PROPIONATE; SALMETEROL XINAFOATE	GLAXO GRP LTD	186			89%	01-Dec-21	26-Aug-26	Device extension
Xopenex	LEVALBUTEROL TARTRATE	SUNOVION			X	33%	08-Oct-24	17-Nov-15	Old device
Spiriva	TIOTROPIUM BROMIDE	BOEHRINGER INGELHEIM	15	13	X	10%	22-Jan-22	12-Mar-27	Device extension
**Epinephrine pens**									
Epipen	EPINEPHRINE	MYLAN SPECLT	139			100%	15-Aug-15	11-Sep-25	Only device patents
**Insulin pens**									
Novolog Flexpen	INSULIN ASPART RECOMBINANT	NOVO NORDISK INC	57	34		60%	20-Dec-17	21-Jul-21	Device extension
Novolog mix Flexpen	INSULIN ASPART PROTAMINE; INSULIN ASPART	NOVO NORDISK INC	168			60%	19-Dec-17	21-Jan-21	Device extension
Lantus	INSULIN GLARGINE RECOMBINANT	SANOFI AVENTIS US	19	16	X	25%	23-Jan-24	23-Mar-28	Device extension
Lantus Solostar	INSULIN GLARGINE RECOMBINANT	SANOFI AVENTIS US	21	11	X	100%	23-Jan-24	12-Apr-25	Device extension
Humalog Kwikpen 100	INSULIN LISPRO RECOMBINANT	LILLY	98	69	X	100%	15-Aug-15	09-Aug-24	Only device patents
Humalog Kwikpen 200	INSULIN LISPRO RECOMBINANT	ELI LILLY AND CO	98	69	X	33%	11-Jun-18	09-Aug-24	Device extension

* Pediatric and other patent extensions are included whenever relevant.

**"Device extension" means the last-to-expire device patent was after that of the last-to-expire formulation patent; "Only device patents" means that only device patents remain unexpired and that all patents of any other type have expired; “Old device" means the last-to-expire patent on the formulation was after that of the last-to-expire device patent; and “No device patents" means that no unexpired device patents were listed. Only patents on the formulation remain unexpired.

Given that the patent system is not likely to change much, whether because of tradition, the political influence of the pharmaceutical industry, or international framework treaties that lay down core requirements for patentability, how should the health system respond to potentially abusive evergreening?

Obviously, the first step is to be alert that device evergreening happens. This paper offers some evidence, but the practice is more widespread than recognized in the FDA Orange Book (hence this paper did not look at insulin pumps, probes, continuous glucose monitors, and other devices which fall outside the Orange Book). One solution would be for FDA to introduce a companion or appendix to the Orange Book which records patents for broader categories of devices that are specifically approved for use with certain medicines (which would capture insulin pumps, for example) or that are part of an integrated system (such as glucose monitors whose software is part of a specific infusion pump). There is probably also scope to apply antitrust law, which prohibits “tied” sales, to the selling of specific medicines only with specific devices, as was done when the Federal Trade Commission prosecuted a pharmaceutical firm for tying its medicine to a specific blood test [24, 25]. Without measures such as these, it falls to health technology assessment and insurance authorities to require suppliers to defend their choice of device technology before making reimbursement decisions.

## Supporting Information

S1 File Raw patent and product information data file. (XLSX)
